# The relationship between a simulated glaucoma impairment and postural threat on quiet stance

**DOI:** 10.1007/s10055-024-01082-0

**Published:** 2025-01-31

**Authors:** Lisa K. Lavalle, Nora Pourhashemi, Taylor W. Cleworth

**Affiliations:** 1 School of Medicine, Queen’s University, Kingston, ON Canada; 2 School of Kinesiology and Health Science, Faculty of Health, York University, Toronto, ON Canada; 3 Centre for Vision Research, York University, Toronto, ON Canada

**Keywords:** Postural control, Virtual reality, Glaucoma, Postural threat

## Abstract

Peripheral visual field deficits developed through glaucoma have been shown to contribute to balance deficits and a fear of falling. Currently, there is no work that examines the relationship between fear of falling and quiet stance among glaucoma patients. Therefore, this study aimed to examine the impact of a virtual height-induced postural threat on balance control among healthy individuals exposed to a simulated glaucoma impairment. Participants stood on a force plate to measure kinetic responses while wearing a virtual reality (VR) head-mounted display (HMD) which also tracked head position. Surface electromyography (EMG) was also used to measure muscle activity from ankle stabilizing muscles. Trials were 60 s, with two at ground level and two at 7 virtual meters above ground, each exposing participants to normal vision and a VR-simulated glaucoma impairment. Electrodermal activity was collected, and questionnaires were completed following each trial to evaluate psychological aspects of the postural threat. Overall, while experiencing height-induced fear with normal vision, participants developed a tighter control of upright stance (decreased amplitude and increased frequency of balance-related movement); however, this was not observed for the simulated glaucoma conditions. Therefore, balance deficits among glaucoma patients may be mediated by fear of falling contributing to an unexpected postural strategy.

## Introduction

To maintain balance control while standing, multisensory integration can combine feedback from the visual, vestibular, and somatosensory systems (Peterka 2002, 2018). Specifically, the visual system plays a crucial role in maintaining upright stance (Bronstein and Buckwell 1997; Lavalle and Cleworth 2023; O’Connell et al. 2017; Redfern et al. 2001). For example, when visual feedback is eliminated by closing the eyes, postural sway during quiet stance can increase by 50% (Diener et al. 1984). Therefore, when visual feedback is compromised, such as through acquired visual disorders, balance deficits can develop. Glaucoma encompasses a group of neurodegenerative visual disorders and is commonly characterized by a gradual visual field loss (Hong and Trope 2015; Weinreb et al. 2014). Without treatment, visual decline often begins at the inferior periphery, affecting the availability of peripheral optic flow (Weinreb et al. 2014). This can be detrimental to postural control as peripheral visual feedback has been shown to be especially sensitive to changes in optic flow and motion detection (Horiuchi et al. 2017; O’Connell et al. 2017). For example, glaucoma patients with greater binocular visual field loss and/or thinner retinal nerve fiber layers have shown a greater degree of sway during upright stance (Black et al. 2008). Additionally, a greater degree of inferior hemifield loss among glaucoma patients has been shown to be related to an increased amplitude of sway during upright stance (Black et al. 2008), as well as a greater standard deviation of ankle torque moments (Diniz-Filho et al. 2015).

Compared to healthy controls, glaucoma patients also report an increased fear of falling (Bhorade et al. 2021; Ramulu et al. 2012). The relationship between fear of falling and balance control is complex. Individuals who are more fearful can demonstrate greater avoidance behaviour, which can increase the risk of developing a sedentary lifestyle and becoming physically deconditioned (Landers and Nilsson 2022). However, heightened state anxiety from fear of falling has also been shown to directly impact balance control while standing (Nielsen et al. 2022; Zaback et al. 2019). Previous work has shown that participants elevated to virtual heights demonstrate an increase in electrodermal activity (EDA) and a decrease in perceived stability and balance confidence (Cleworth et al. 2012; Nielsen et al. 2022). It was further observed that this experience of postural threat led to individuals developing a stiffening strategy, as demonstrated by decreased amplitude and increased frequency of centre of foot pressure (COP) (Cleworth et al. 2012).

Currently, there is no work that examines fear of falling while glaucoma patients are participating in a balance task. For example, one study asked patients to rate how fearful they predicted themselves to be while conducting certain tasks of daily living (Daga et al. 2017). Although a significant correlation was established between self-rated fear of falling and perceived balance abilities (Daga et al. 2017), an objective relationship between fear of falling and balance control has yet to be studied. Virtual reality (VR) demonstrates a promising way to study this relationship. Recently, VR has been successfully used to simulate disorders affecting the visual field (Jones and Ometto 2018; Jones et al. 2020); this allows for the functional impact of these visual disorders to be studied in a controlled environment. By using virtual environments that are photo-realistic, previous work has also shown VR to demonstrate adequate behavioural realism (Riecke et al. 2006).

Therefore, the aim of this study was to assess the relationship between postural threat and quiet stance among individuals with a VR-simulated glaucoma impairment. It was hypothesized that while standing at an increased virtual surface height, participants would develop a stiffening strategy as evidenced by a decreased amplitude and increased frequency of balance-related movement, and greater agonist/antagonist co-contraction when standing at height with normal vison. In addition, sample entropy was hypothesized to increase, as it has been thought to resemble an increase in automatic control of upright stance (Fischer et al. 2023). Given that glaucoma patients demonstrate an increase in postural sway compared to healthy controls, yet sample entropy related changes are not clear, it was unclear whether this same stiffening strategy with changes in control strategies would be present with the glaucoma simulation at height. Lastly, it was hypothesized that while at height, changes related to fear and anxiety would be greater among the simulated glaucoma conditions.

## Methods

### Participants

Thirty-one healthy adults (mean age (SD): 22.9 (3.0) years; 20 female) volunteered to participate in this study from York University and the surrounding community. To be eligible for participation, individuals were required to be between 18–40 years old and have no known orthopedic or neurological impairments that could influence balance. All participants provided written, informed consent in accordance with the Human Participants Review Sub-Committee of York University’s Ethics Review Board prior to participation (#e2022-064).

### Experimental set-up

**Fig. 1:**
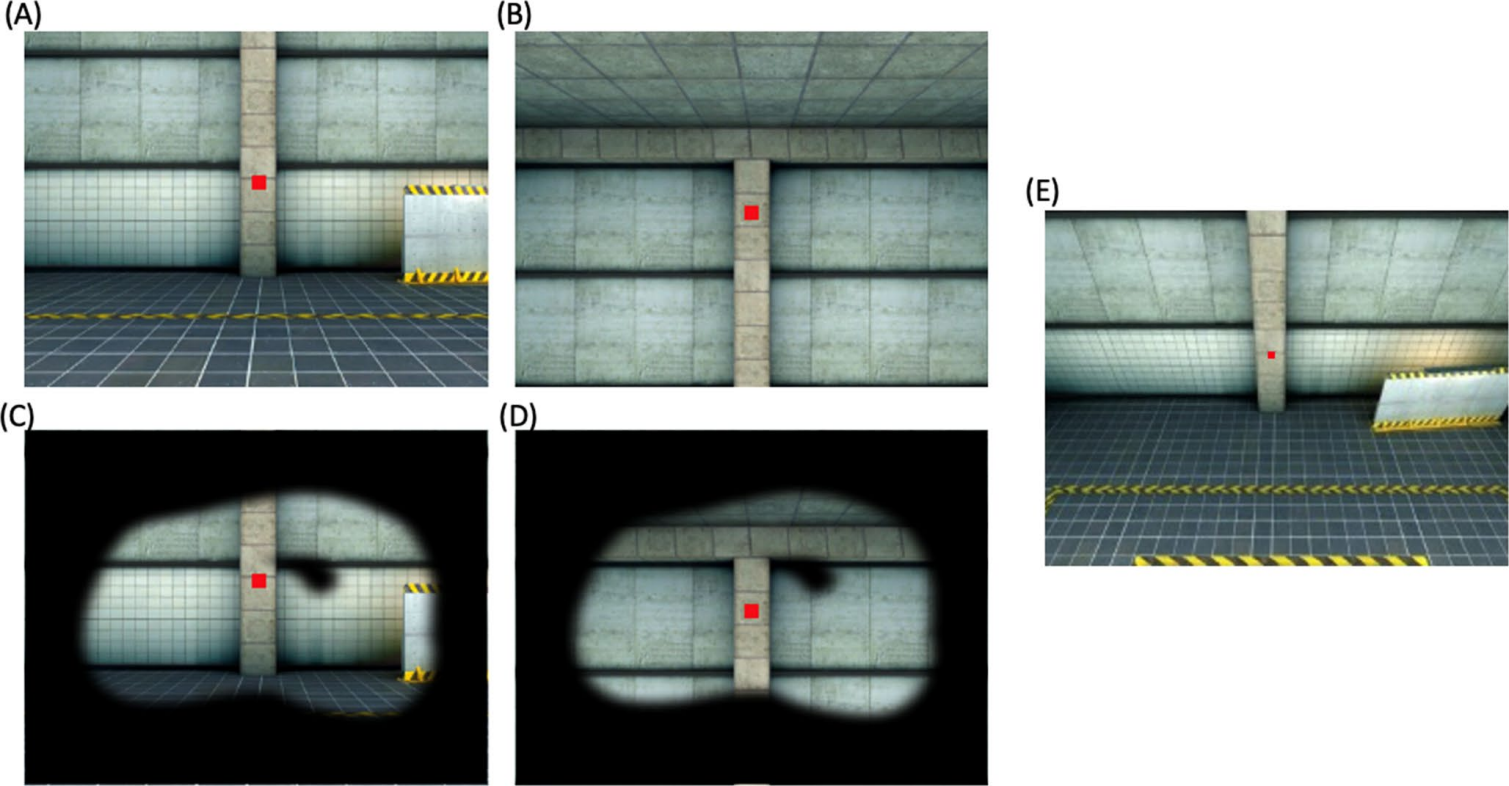
Participants’ view within the VR scene for each of the 4 conditions (**A**–**D**), and while looking down at height during the second immersion protocol (**E**). The glaucoma simulation is shown for both the Low (**C**) and High (**D**) conditions. **A**, **B** show the view with normal vision at Low (**A**) and High (**B**) height. Each of the 4 conditions show a red target ahead of the participant for them to focus on during the trials

Participants stood on a force plate (AMTI, USA) capable of capturing ground reaction forces and moments while wearing a VR head-mounted display (HMD) (HTC Vive: up to 120 degrees horizontal field of view). A photorealistic VR environment, “pit.osgb” (developed by WorldViz, USA) was modified to include a virtual platform that rose to 7 m above ground and contained red targets at eye level for participants to focus on during the trials (Fig. 1). EDA was recorded from the right thenar and hypothenar eminences (Skin Conductance Module 2502, Cambridge Electronic Design (CED), UK). Surface electromyography (EMG) electrodes were placed on the right tibialis anterior (TA), right medial gastrocnemius (MGas), and right soleus (Sol) muscles (Ultium, Noraxon, USA). Maximum voluntary contraction (MVC) protocols were completed for each of the three muscles. Participants completed dorsiflexion under resistance for TA, plantarflexion under resistance with the knee angled at approximately 90° for Sol, and plantarflexion under resistance while lying prone for MGas.

### Procedure

There was a series of four trials: two at ground level and two at 7 virtual meters above ground, each exposing participants to normal vision and a simulated glaucoma impairment (Fig. 1). For each trial, participants aligned the front of their feet with the front edge of the force plate, with feet placed together. Foot position was then marked with tape to ensure consistent placement across trials. Participants were asked to stand quietly with their arms rested naturally beside their trunk, while focusing on the red target ahead of them in the VR scene. Both trials at ground level were completed first to maximize height effects (Adkin et al. 2000), followed by the trials at height. The order of visual conditions was counterbalanced for each height and participant. Prior to the first trial at ground level, participants completed an immersion protocol, where they had to locate three red squares in the virtual environment. The HMD was kept on for the duration of the trials to maintain immersion within the virtual environment, including when being raised to the virtual height. Prior to completing the first trial at height, participants were instructed to shift their feet forward to feel the edge of the force plate which aligned with the edge of the platform in the VR scene. Then, they were asked to look down and acknowledge that they perceived themselves to be at the edge of the virtual platform. After each trial, subjective reports of balance confidence, fear of falling, perceived stability, and state anxiety were assessed by having participants answer a series of questionnaires projected in the VR scene. Specifically, participants were asked to rate balance confidence, fear of falling, and perceived stability on a visual analog scale ranging between 0–100, with 100 being greatest confidence, fear, and perceived stability. State anxiety was assessed through a series of 16 questions, each using a 9-point Likert scale, which were summed to produce a total anxiety score (maximum score of 144 indicated the most anxiety).

### Measures

Ground reaction forces and moments were sampled from the force plate at 100 Hz (Power 1401 with Spike2 software, CED, UK). COP was calculated in MATLAB (R2022b, Mathworks Inc., USA) in the anteroposterior (AP) and mediolateral (ML) directions. COP was low pass filtered at 5 Hz using a second-order dual-pass Butterworth filter and underwent bias removal by subtracting the mean COP position. Head displacement (HD) was captured in the AP direction through the HMD, outputted through Vizard using a digital lab interface (LabJack, Colorado, USA), and recorded at 100 Hz (Spike2, CED, UK). HD was low pass filtered with a 5 Hz second-order dual-pass Butterworth filter and underwent bias removal by subtracting the mean HD position from the signal. Sample entropy (SE), root mean square amplitude (RMS), and mean power frequency (MPF) were calculated in MATLAB for AP and ML COP and AP HD. EDA activity was sampled at 100 Hz, filtered using a 1 Hz fifth-order dual-pass Butterworth filter, and then converted to microsiemens (μS). Mean EDA was then calculated in MATLAB. The sum of scores across each questionnaire were totaled. EMG data was acquired and recorded by a Noraxon Ultium wireless EMG system sampled at 2000 Hz and bandpass filtered 5 to 500 Hz. EMG data was then converted into an analog signal and sent to the data acquisition device (Power 1401) to synchronize with other analog data (force plate, HD, EDA) and sampled at 2000 Hz (CED). EMG signals were then processed in MATLAB which included a 30–500 Hz bandpass filter, bias removed, rectified, normalized to %MVC, and low passed filtered at 3 Hz to create a linear envelope. EMG outcome measures were calculated in MATLAB and included mean normalized EMG and co-contraction indices (CCI) (TA/SOL, TA/MGas). CCIs were calculated by computing the point-by-point minimum values between the normalized EMG signals of the agonist and antagonist muscles for the duration of each trial. These values were then integrated using the trapezoidal rule and were normalized by the length of the trial to compute the final CCI.

### Statistical analysis

5 participants were excluded from EDA analysis and 3 from EMG analyses due to technical errors during data collection. Due to the robust nature of repeated measures analysis of variance (ANOVA) tests, 2 (height: Low, High) × 2 (visual condition: Normal, Glaucoma) repeated measures analysis of variance (ANOVA) tests were conducted in SPSS (IBM Corp., N.Y., USA) for all outcome measures (19 in total) despite violations of normality. Statistical significance was set at an α-level of 0.05 and Sidak corrections were applied to post hoc comparisons to correct for multiple comparisons within an outcome measure. Outliers were identified using boxplots and, for kinetic and kinematic measures, by plotting time series data in MATLAB. 12/744 (1.6%) of COP/HD, 3/104 (2.9%) of EDA, and 18/548 (3.3%) of EMG data points were identified as outliers. Identified outliers were replaced to three standard deviations from the mean (Field 2009). Outliers for questionnaire data were not corrected. Normality was assessed using Shapiro-Wilks tests and histograms. Non-parametric t-tests were used post-hoc to explore significant interaction effects where normality was violated.

## Results

### EDA and questionnaire data

**Fig. 2:**
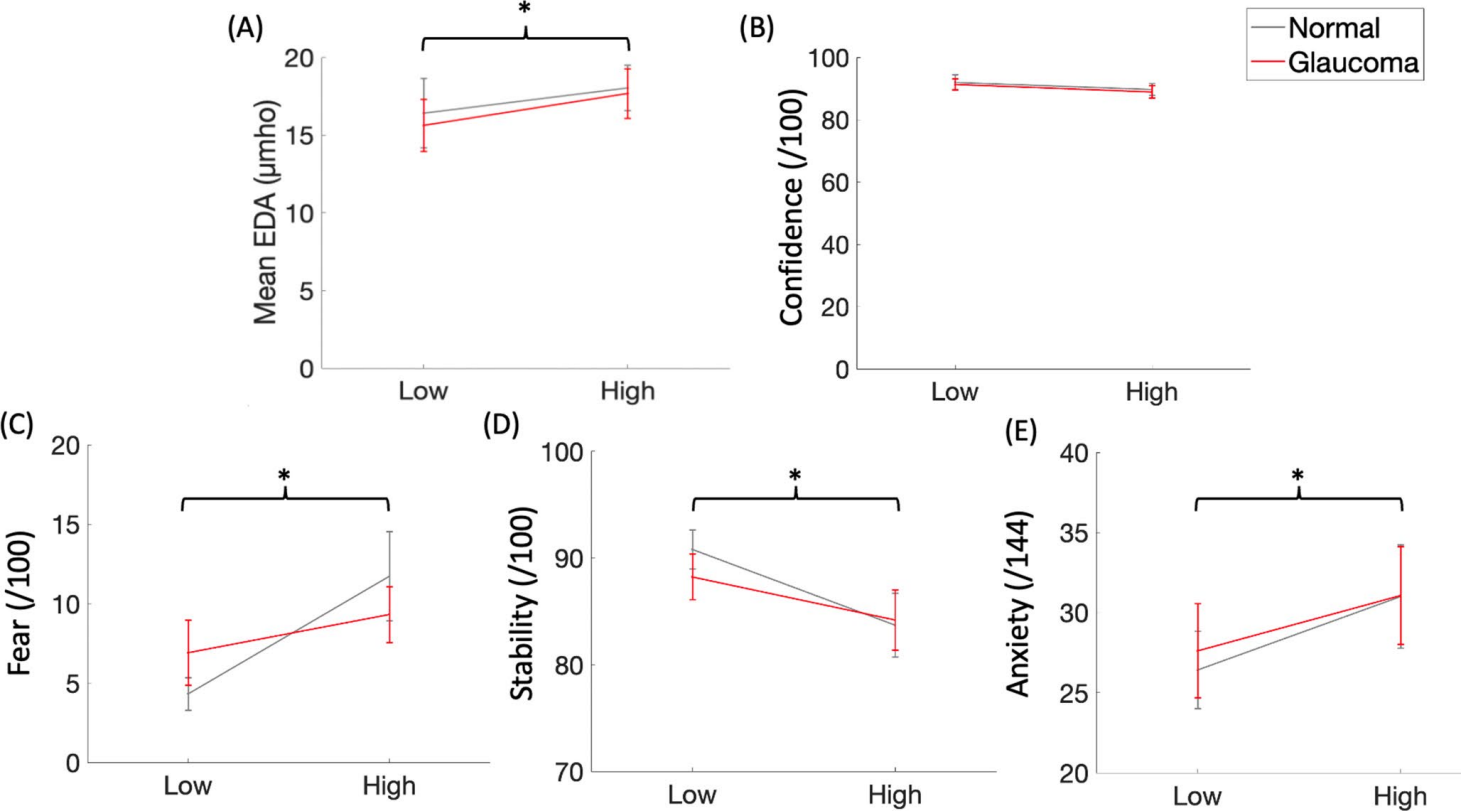
Mean values (+ / − 1 standard error) for **A** EDA, **B** Balance Confidence **C** Fear, **D** Perceived Stability, and **E** Anxiety plotted across low and high heights for normal (grey) and simulated glaucoma (red) conditions. Black brackets indicate main effects of height

For mean EDA, there was a significant main effect of height, with no significant main effect of vision or a significant interaction effect observed (Table 1). Mean EDA was greater at height, compared to ground level (Fig. 2). There was a significant main effect of height on fear of falling, perceived stability, and anxiety (Table 1). Fear and anxiety were greatest at height, while perceived stability decreased with an increase in height (Fig. 2). There was no significant main effect of height on balance confidence observed (Table 1). There were no significant main effects of vision or any significant interaction effects observed for any of the questionnaire outcome measures (Table 1).

**Table 1:** Main effects and interaction effects displayed from repeated measures ANOVA tests for COP, HD, EMG, psychological, and arousal outcome measures

			Height		Vision		Height*Vision	
			F_(1,30)_	p-value	F_(1,30)_	p-value	F_(1,30)_	p-value
COP & HD	AP COP	RMS	0.008	0.931	0.170	0.683	**6.576**	**0.016**
		MPF	1.788	0.191	0.863	0.360	**4.806**	**0.036**
		SE	2.561	0.120	0.099	0.755	**7.927**	**0.009**
	ML COP	RMS	0.693	0.412	0.151	0.700	0.163	0.690
		MPF	2.057	0.162	0.183	0.672	0.096	0.759
		SE	**8.284**	**0.007**	0.003	0.956	0.280	0.601
	AP HD	RMS	0.010	0.921	0.058	0.812	**5.138**	**0.031**
		MPF	0.320	0.576	3.453	0.073	1.015	0.322
		SE	1.024	0.320	0.970	0.333	**4.915**	**0.034**

			F_(1,27)_	p-value	F_(1,27)_	p-value	F_(1,27)_	p-value
EMG		% MVC TA	1.371	0.253	0.071	0.792	0.473	0.498
		% MVC SOL	1.084	0.307	0.414	0.526	0.003	0.956
		% MVC MGas	0.370	0.548	0.192	0.664	0.010	0.920
		CCI TA/Sol	1.930	0.177	0.016	0.899	0.081	0.778
		CCI TA/MGas	1.316	0.262	0.668	0.422	0.069	0.796

			F_(1,30)_	p-value	F_(1,30)_	p-value	F_(1,30)_	p-value
Psychological Measures		Confidence	1.811	0.188	0.740	0.396	0.017	0.898
		Stability	**7.885**	**0.009**	0.838	0.367	1.681	0.205
		Fear	**7.551**	**0.010**	0.107	0.745	1.608	0.214
		Anxiety	**8.236**	**0.007**	0.553	0.463	0.472	0.497

			F_(1,25)_	p-value	F_(1,25)_	p-value	F_(1,25)_	p-value
Arousal		EDA	**4.898**	**0.036**	0.220	0.643	0.450	0.508

Bolded values denote statistical significance.

### COP and HD data

**Fig. 3:**
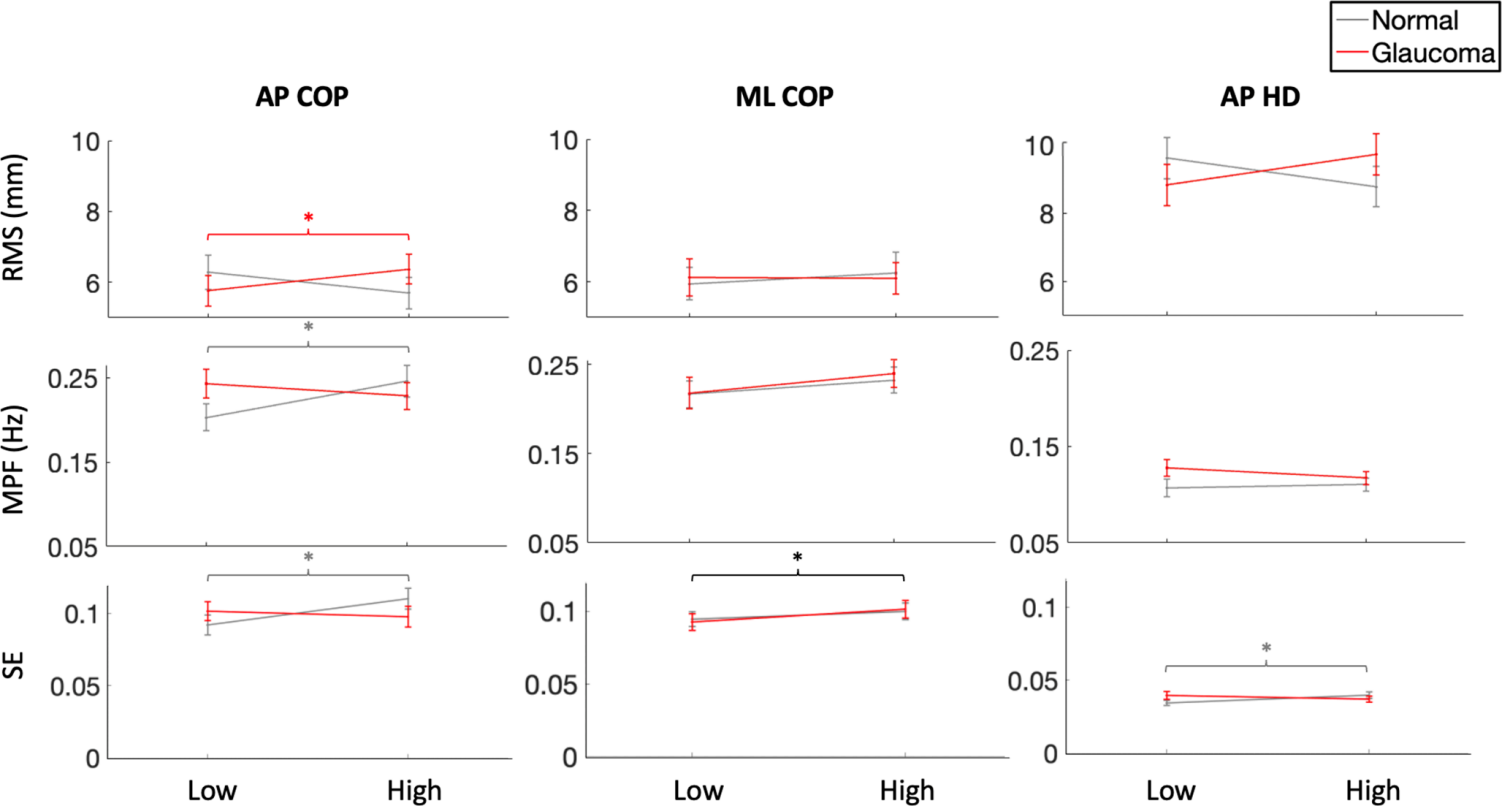
Mean values (+ / − 1 standard error) for COP and HD RMS, MPF, and SE plotted across low and high heights for normal (grey) and simulated glaucoma (red) conditions. Black brackets indicate main effects of height. Significant post-hoc tests displayed with grey brackets for normal vision and red brackets for simulated glaucoma

There was a significant main effect of height on ML COP SE (Table 1), where SE was greatest at height (Fig. 3). There were no other significant main effects of height or significant main effects of vision on COP and HD outcome measures (Table 1). There were significant interaction effects observed for AP COP RMS, MPF, and SE (Table 1). With simulated glaucoma, AP COP RMS was greatest at height (t(30) = 2.02, p = 0.044), with no significant change with normal vision (t(30) = 1.67, p = 0.096). AP COP MPF and AP COP SE were greatest at height with normal vision (MPF: t(30) = 2.31, p = 0.021; SE: t(30) = 2.67, p = 0.008), with no significant change observed with simulated glaucoma (MPF: t(30) = 0.94, p = 0.347; SE: t(30) = 0.92, p = 0.357). There were also significant interaction effects observed for AP HD RMS and SE (Table 1). Post-hoc analyses showed AP HD RMS had no significant change between low and high heights with either normal vision (t(30) = 1.84, p = 0.065) or simulated glaucoma (t(30) = 1.74, p = 0.081). AP HD SE was greatest at height with normal vision (t(30) = 2.21, p = 0.027), but not simulated glaucoma (t(30) = 1.14, p = 0.256). No other significant interaction effects were observed for the other COP and HD outcome measures (Table 1).

### EMG data

**Fig. 4:**
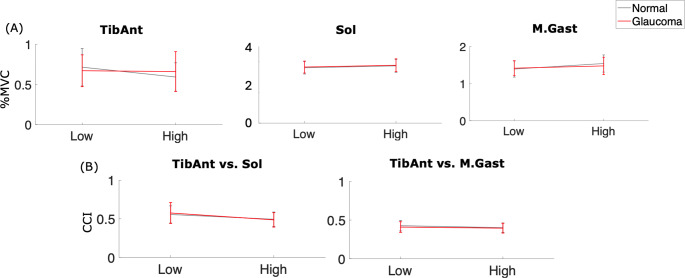
Mean values (+ / − 1 standard error) for **A** % MVC activation for TA, Sol, and MGas muscles and **B** CCI for TA/Sol and TA/MGas plotted across low and high heights for normal (grey) and simulated glaucoma (red) conditions

There were no significant main effects of height or vision, or a significant interaction effect observed for TA, Sol or MGas mean % MVC activity (Fig. 4, Table 1). In addition, the CCI for TA/MGas and TA/Sol showed no significant main effects of height or vision and no significant interaction effect (Fig. 4, Table 1).

## Discussion

The aim of this study was to use a height-induced postural threat to examine the relationship between fear of falling and simulated glaucoma on quiet stance. Overall, the results confirmed that standing at a virtual height of 7 m elicits a postural threat (Adkin et al. 2002; Nielsen et al. 2022; Sibley et al. 2007). Further, the visual field deficit created through the glaucoma simulation was shown to compromise the expected height-induced strategy, where a decrease in amplitude and increase in frequency of balance-related movement no longer occurs while experiencing a postural threat.

While standing at a virtual height, there was a significant increase in mean EDA independent of the visual condition. This means that while exposed to both normal vision and simulated glaucoma, participants experienced increased arousal. Previous work has shown there to be an increase in skin conductance and EDA when participants stood at elevated surface heights (Adkin et al. 2002; Nielsen et al. 2022; Sibley et al. 2007). This relationship has been called “postural anxiety”, as there is a direct correlation between increased skin conductance and psychological state anxiety (Lader 1983). In this study, an increase in EDA with height was complemented by subjective measures of postural threat. At height, there was a significant increase in fear and anxiety, and a decrease in perceived stability, independent of visual condition supporting the conclusion that the 7 m virtual height was effective at eliciting a postural threat when the field of view is reduced. The psychological effects of height-induced threat also appeared to be similarly experienced between normal vision and simulated glaucoma, with no effect of simulated glaucoma on these responses in the Low threat condition. It has been well-reported that individuals with glaucoma experience an increased fear of falling at baseline compared to the healthy population (Bhorade et al. 2021; Ramulu et al. 2012). However, this study assessed fear of falling in response to a situational task (i.e. being raised to height), rather than fear during activities of daily living. Since the participants fixed their gaze forward during the trials, it is possible that the restricted field of view with simulated glaucoma reduced their ability to adequately assess the level of threat and their proximity to the platform and ground. This could contribute to both visual conditions demonstrating similar fear at height, despite the simulated glaucoma reducing the field of view.

In addition to linear measures of balance control, SE was calculated as a non-linear measure to assess the regularity of COP and HD time series data. A higher SE indicates more irregularity in the data, meaning that there is a lower probability of data sequences repeating (i.e. the value of one data point does not depend on the value of previous points) (Fischer et al. 2023; Richman and Moorman 2000). In the literature, it has been suggested that one distinguishing factor between high and low COP SE could be the degree of cognitive involvement required in a balance task (Roerdink et al. 2011; Uiga et al. 2018). For example, stroke patients have been shown to have a lower COP SE than healthy controls, as increased cognitive demands are required to overcome neuromuscular restrictions (Roerdink et al. 2006). Following stroke rehabilitation, SE has been shown to increase, suggesting that less cognitive resources are required for balance control (Roerdink et al. 2006). The results of this study showed that in the AP direction, COP and HD SE increased with normal vision while at height. In the context of previous literature, this would suggest that with normal vision, there is greater automaticity within the postural control strategies used while experiencing a height-induced threat. It is therefore possible that individuals with peripheral field deficits, such as glaucoma patients, respond to postural threat by diverting more cognitive attention to the balance task, rather than relying on automatic strategies. In the ML direction, SE increased regardless of the visual condition. The postural threat in this study (i.e. falling off the platform ledge) was presented in the AP plane as participants’ gaze was fixed forwards during the immersion protocol and throughout the trials. Therefore, it is possible that less cognitive demands were used by both visual groups to control ML COP, thus, increasing SE. However, recent work has also shown SE to increase with an increase in postural threat (Ellmers et al. 2021; Fischer et al. 2023), suggesting that threat may enhance the automaticity of the postural strategy. It has also been suggested that greater conscious processing of balance control may hinder this relationship, compromising the postural strategy (Ellmers et al. 2021). In the context of this study, the visual deficit could have introduced an element of conscious attention to the balance task, reducing the automaticity of the sway response.

With normal vision, AP COP MPF increased with an increase in surface height. Previous work has suggested that an increased frequency of COP displacements could be related to increased ankle moments being exerted to keep the center of mass within a smaller area in the base of support, thus, creating a stiffening strategy (Schinkel-Ivy et al. 2016; Sweeny et al. 2021). This strategy was to be expected at height, as it has been observed that individuals develop a tighter control of upright stance when faced with a height-induced postural threat (Adkin et al. 2000; Carpenter et al. 1999; Cleworth et al. 2012; Davis et al. 2009). Previous work has also shown that individuals make postural adjustments in the same direction that they perceive the threat as a way to limit movement in that direction (Carpenter et al. 2001; Raffegeau et al. 2020). As mentioned above, if participants in this study perceived the threat of falling off the platform in the AP plane, this could explain why COP MPF adjustments were significant in the AP, rather than ML direction. Additionally, the results of this study show that AP COP RMS was significantly greater at height for the glaucoma simulation only. Previous work has shown that individuals who are exerting more conscious control of balance at height lean further away from the platform edge (Huffman et al. 2009), resulting in an increased RMS in the AP direction. Therefore, the results from the current study using linear (RMS) and non-linear (SE) analyses suggest that the glaucoma simulation may have led participants to use more cognitive resources to maintain balance control, especially during postural tasks with increased levels of fear and anxiety.

Among EMG measures, there were no differences between heights or visual conditions (Fig. 4) despite changes in COP and HD. While the TA causes ankle dorsiflexion and is active when holding a static position posteriorly or causing an anterior acceleration in postural sway, and the Sol and MGas are responsible for plantarflexion with increased activation when holding an anterior leaning position or during posterior acceleration, they were not influenced by height or glaucoma simulation. Prior results have indicated virtual height causes a smaller effect on COP mean position when standing at height compared to real heights (Cleworth et al. 2012). A smaller shift away from the edge would suggest limited effects on mean EMG activity. However, given the changes in movement-based data between heights and/or visual conditions, and no change in muscle activity across conditions, further research is needed to identify the strategy used during visual decline and postural threat, especially in a virtual environment (Fig. 4).

### Limitations

The current study simulated glaucoma using VR and an HMD. One limitation of this system is the reduced field of view in normal conditions, prior to any visual deficit simulations. While observing a scene through an HMD, peripheral optic flow is reduced compared to real environments. However, when comparing quiet stance with and without the use of an HMD, limited effects on balance control have been observed when realistic environments are presented (Assländer and Streuber 2020). Lastly, while at height, the peripheral field deficit in the glaucoma simulation could have made it challenging for participants to recognize that they were standing at height. With a restricted field of view through the simulation, they could only see the wall ahead of them, making it difficult to gauge their distance to the ground or platform edge during the trials. This limitation was lessened through the immersion protocol participants completed while at height, where they looked down to see the platform edge, and heard the sound of a mechanical hoist while being raised to height. Since conditions were not counterbalanced between participants, there is a possibility of an order effect. All participants completed the Low Threat conditions before High Threat, as prior experience to postural threat may affect postural control even in the absence of the threat (Adkin et al. 2000). Lastly, this study was conducted on healthy adults, where the peripheral field deficit was introduced suddenly to those with normal vision. Vision loss through glaucoma can be a gradual process (Salazar et al. 2020). Therefore, while applying these results to a clinical population, it is important to recognize that some patients may be able to compensate for this loss through sensory re-weighting over time.

## Conclusions

In conclusion, this study showed that height-induced postural threat impacts balance control differently among normal vision and VR-simulated glaucoma. Although standing at height impacted the psychological measures of both visual groups similarly, only individuals with normal vision developed a tighter control of upright stance while under postural threat. Future research is needed to understand how this relationship translates into tasks of dynamic stance and functional tasks of daily living. By understanding how the relationship between fear of falling and balance control impacts glaucoma patients, fall prevention programs can be appropriately tailored for those with peripheral field deficits.

## Data Availability

The datasets generated and analyzed during the current study are available from the corresponding author upon reasonable request.
